# Supplementary material for: Animal Venom for Medical Usage in Pharmacopuncture in Korean Medicine: Current Status and Clinical Implication
Source: Toxins (Basel). 2021 Feb 1;13(2):105. doi: 10.3390/toxins13020105 (PMC7912904; doi:10.3390/toxins13020105)
Supplement: Supplementary file 1 [file toxins-13-00105-s001.pdf]

Supplementary Materials: Animal Venom as a Medical Usage in Pharmacopuncture in Korean Medicine: Current Status and Clinical Implication

Soo-Hyun Sung, Ji-Won Kim, Ji-Eun Han, Byung-Cheul Shin, Jang-Kyung Park and Gihyun Lee

Additional file 1. Demographics of pharmacopuncture-EHDs.

Characteristics	P-EHD<br>*n* (%)
No. of EHD	11 (100.0)
TKM clinics	8 (72.7)
TKM hospital	3 (27.3)
Location	
Seoul	3 (27.3)
Incheon/Gyeonggi province	3 (27.3)
Daejeon/Sejong/Chungcheong province	–
Gangwon province	1 (9.0)
Gwangju/Jeolla province	–
Daegu/Gyeongbuk province	2 (18.2)
Pusan/Gyeongnam province	2 (18.2)
Number of pharmacopuncture	Average 18.4

Additional file 2. Current status of external herbal dispensaries preparing pharmacopuncture: a survey.

Managing Department

Ministry of Health and Welfare

Agent in Charge of the Survey

NIKOM

[Objectives]

By collecting data on the current preparation status of pharmacopuncture of external herbal dispensaries (EHDs), we intend to provide evidence for the development of a safety management plan/policy for the pharmacopuncture of EHDs.

[Targets]

All community health centers in Korea

[Items]

The survey items comprised two parts:
① Basic status: Preparation status; location; type of pharmacopucnture;
② Preparation status of pharmacopuncture: pharmacopuncture name; composition of pharmacopuncture; amount of preparation (vial).

[Instructions]

The Ministry of Health and Welfare intends to conduct a survey to examine the current preparation status of pharmacopuncture of EHDs. Your answers will contribute to the establishment of a safety management plan/policy for pharmacopuncture. The contents of this questionnaire will be used in statistical analysis for academic purposes only, and any personal information will remain strictly confidential other than that which is used for statistical analyses.

[PART 1. Demographic Information]

1. Are you preparing pharmacopuncture in the EHDs?

① Yes
② No

2. Where is your affiliated external herbal dispensary located?

① Seoul	② Busan	③ Incheon	④ Daegu	⑤ Gwangju	⑥ Daejeon	⑦ Ulsan	⑧ Sejong
⑨ Gyunggi	⑩ Gangwon	⑪ Chungbuk	⑫ Chungnam	⑬ Jeonbuk	⑭ Jeonnam		
⑮ Gyeongbuk	⑯ Gyeongnam	⑰ Jeju					

3. What type of pharmacopuncture is to be prepared?: (______________Types)

4. What category does the TM institution with the EHD belong to?

① Traditional Korean medicine clinics (primary healthcare institution)
② Network Traditional Korean medicine clinics (primary healthcare institution)
③ Traditional Korean medicine hospital (Secondary healthcare institution)
④ Public health center
⑤ Other: _______________

[PART 2. Preparation Status of Pharmacopuncture]

NO.	Pharmacopuncture Name	Composition of Pharmacopuncture	Amount of Preparation (Vial)
1			
2			
3			
4			
5			
6			
7			
8			
9			
10			
...			
